# Dr. James H. Haley

**Published:** 1887-03

**Authors:** 


### NECROLOGICAL.

### OBITUARY.

JAMES H. HALEY, M. D., died, very suddenly, at his residence in Moffett, Bell county, Texas, about 6 o'clock Sunday morning, the 20th February, 1887, doubtless of some heart and lung trouble

Dr. Haley was born in Carroll county, Miss., Nov. 5th, 1831. His father moved to Madison county when the son was quite a youth. Here he remained until he entered college. Dr. Haley graduated in medicine from Jefferson Medical College, Pa., the 12th March, 1856. After receiving his diploma from this world-renowned institution, he returned to Jackson, Miss., where he entered the Lunatic Asylum, as one of the attending physicians, and remained in this charity for two years. While a resident of Jackson, he was united in marriage, to Miss Cora Alford, who, with eight children, survives him.

Dr. Haley was a Surgeon in the P. A. C. S., and was at the never-to-be-forgotten seige of Vicksburg.

Since the close of the war, he has been regularly engaged in the practice of his chosen profession. He moved to Texas, in January, 1870, residing in Washington county about four years. In the autumn of 1873, he moved to Moffett, Texas, where he lived to the hour of his death.

Dr. Haley was one of the original members of the Bell County Medical Association, and was twice its presiding officer, having received a unanimous endorsement on both occasions of his election. He was always in attendance when possible, riding a distance of thirteen miles, and frequently in inclement weather, that he might aid and assist in maintaining and building up the Society, of which he was always proud.

Dr. Haley was a consistent member of, and a shining light in, the Methodist Episcopal Church, of which he had been a worthy communicant for over thirty years.

It is a sad and painful task to chronicle the death of such a man as Dr. James H. Haley. Truly his death has overshadowed the entire community in which he lived, with gloom and sorrow. He will be missed in the Church, the State, in the profession, in the Medical Society. He will be missed as a neighbor, as a friend, as a citizen. He will be missed among the sick and the distressed of the section where he so long and so successfully practised his vocation. But, above all, how sadly missed around the family altar! None can tell, not even a broken-hearted and disconsolate bosom companion.

The writer saw him on Thursday before his death on Sunday, and though he was deeply impressed with his physical appearance and failure, had no thought of death coming so soon to his relief.

The day before his death, he visted a patient in the country, returning home late in the evening through a very chilly and damp atmosphere. About two weeks prior to this time, he came very near his death, by a sudden and severe attack of asthma, attended, as he informed me, by more congestion of the lungs than is common, perhaps, in this disease. On Saturday night, after a light supper, he read to his family and retired to bed about 9 o'clock, in better spirits than he had been, since the severe attack, before alluded to. On Sunday morning he awoke his little son to kindle a fire. A few minutes after this, his daughter heard a strange noise and singular struggle, and on rushing into his room found her father in the last agonies of death. His immortal spirit was just leaving "this tenement of clay," and returning "to God who gave it." On Monday, his mortal remains were conveyed to their last resting place, by the hands of friendship and love, followed by one of the largest funeral processions ever formed in Bell county. He was buried by the Knights of Honor, of which organization he was a member in good standing.

Dr. Haley was no ordinary man. In all the virtues that go to make up and adorn the true man and gentleman, Dr. Haley was without a superior. He possessed, in an eminent degree, all the characteristics of a faithful follower of the Good Samaritan.

The writer of this brief and imperfect notice, knew the deceased well, having first formed his acquaintance during the session of 1855 and '56, at Jefferson Medical College. In the bloom of youth they sat together on the same benches, for one long, though pleasant, session.

Dr. Haley had as few faults as any man within his knowledge. He was absolutely above a low, mean, contemptible act. His thoughts and aspirations ran in higher and purer channels. He was fair, honest, open, frank, kind, generous, courteous, obliging; in short, the very embodiment of *truth* and *honor*.

All who knew him loved him, and will ever revere his sacred memory.

"His life was gentle, and the elements so mixed in him, that nature might stand up and say to all the world, *this was a man*."

